# Why Bacteriophage Encode Exotoxins and other Virulence Factors

**Published:** 2007-02-28

**Authors:** Stephen T. Abedon, Jeffrey T. LeJeune

**Affiliations:** 1 Department of Microbiology, Ohio State University, Mansfield, Ohio; 2 Food Animal Health Research Program, Ohio State University, Wooster, Ohio

**Keywords:** Exotoxins, Virulence Factors, Phage, Bacteriophage

## Abstract

This study considers gene location within bacteria as a function of genetic element mobility. Our emphasis is on prophage encoding of bacterial virulence factors (VFs). At least four mechanisms potentially contribute to phage encoding of bacterial VFs: (i) Enhanced gene mobility could result in greater VF gene representation within bacterial populations. We question, though, why certain genes but not others might benefit from this mobility. (ii) Epistatic interactions—between VF genes and phage genes that enhance VF utility to bacteria—could maintain phage genes via selection acting on individual, VF-expressing bacteria. However, is this mechanism sufficient to maintain the rest of phage genomes or, without gene co-regulation, even genetic linkage between phage and VF genes? (iii) Phage could amplify VFs during disease progression by carrying them to otherwise commensal bacteria colocated within the same environment. However, lytic phage kill bacteria, thus requiring assumptions of inclusive fitness within bacterial populations to explain retention of phage-mediated VF amplification for the sake of bacterial utility. Finally, (iv) phage-encoded VFs could enhance phage Darwinian fitness, particularly by acting as ecosystem-modifying agents. That is, VF-supplied nutrients could enhance phage growth by increasing the density or by improving the physiology of phage-susceptible bacteria. Alternatively, VF-mediated break down of diffusion-inhibiting spatial structure found within the multicellular bodies of host organisms could augment phage dissemination to new bacteria or to environments. Such phage-fitness enhancing mechanisms could apply particularly given VF expression within microbiologically heterogeneous environments, ie, ones where phage have some reasonable potential to acquire phage-susceptible bacteria.

Viruses are either the simplest of living things or are among the more complex of non-living things. At the very least, viral gene pools can form a continuum with those of their hosts, with gene exchange and recombination occurring between viral and host DNA. This gene exchange can be so pervasive that the viruses of bacteria—known as phages or bacteriophages—have been suggested as substantial or even the most significant drivers of bacterial evolution (Krisch 2003). So-called temperate phage appear to play particularly important roles in bacterial evolution since, as prophage, they are able to establish long-term genetic symbioses with their hosts, typically by the phage genome directly (though reversibly) integrating into the host chromosome. Together these prophage-bacterial combinations are described as lysogenic bacteria or, simply, as lysogens.

Numerous bacterial virulence factors (VFs)—agents known to contribute to the development of infectious disease in eukaryotes such as ourselves—have been shown to be encoded by prophage (Brüssow et al 2004). Human diseases directly caused by prophage-encoded VFs include (but are not limited to) botulism, diphtheria, cholera, and those associated with Shiga toxigenic *Escherichia coli* such as *E. coli* O157 (Boyd et al 2001; Boyd and Brüssow 2002; Brüssow et al 2004; Boyd 2005). Indeed, the defining exotoxin for each of the listed diseases—respectively botulism toxin, diphtheria toxin, cholera toxin, and Shiga toxin—is expressed from a phage-encoded gene. The emergence and progression of many bacterially mediated infectious diseases consequently are affected by temperate-phage movement between bacteria (Breitbart et al 2005) as well as by prophage-mediated control of VF production (Wagner and Waldor 2002).

Though phage encoding of virulence-factor genes is important to an understanding of many bacterially mediated infectious diseases, here we will consider a more fundamental issue: Why do phage, particularly prophage, even encode VFs? In principle there exist at least four entities that could selectively benefit from the resulting association: the VF gene, the bacterium host, bacterial populations, and the encoding phage/prophage. Furthermore, a given VF-associated phenotype could simultaneously benefit any or all of these four entities. We list in Table 1 the various mechanisms considered here which could contribute to the maintenance of prophage-VF associations. Of those ten mechanisms, we note that only one conceivably benefits individual VF-expressing bacteria (mechanism 6: Epistasis linking VF and phage genes). By contrast, five of our proposed mechanisms instead benefit particularly the VF-expressing gene (mechanisms 1–5) while two or three (depending on the system) benefit the bacterial population (ie, rather than individual, VF-expressing bacteria; mechanisms 7 and 8, plus 6 for some systems). Alternatively, benefits may be accrued by VF-gene harboring phage (mechanisms 9 and 10).

The order in which we discuss these mechanisms reflects our bias in considering especially the *virions* of lytic phage to be distinct organisms. That is, as bacterial predators, parasites, or horizontally transmitted entities, the virions of lytic phage, even of temperate phage, exist at least semi-autonomously from their bacterial hosts. Such a perspective— which we believe may be key to understanding why temperate phage appear to commonly encode bacterial VFs—contrasts a viewing of phage (especially prophage) instead as somewhat integral components of various bacterial pathogens. In terms of levels of selection, we therefore order our discussion of the mechanisms contributing to phage maintenance of bacterial VF genes by going from gene to prophage to individual bacterium to bacterial population, but only subsequently, and separately, do we discuss selection acting upon phage virions. Thus, we consider a three-organism/four-component system—phage, bacterial host, eukaryotic host, and VF—where VFs are active against eukaryotic hosts, are expressed within and by bacteria, and are horizontally transmitted between bacteria by the phage virions produced by induced prophage.

Where appropriate we extend our emphasis to include non-phage mobile genetic elements (MGEs; Davis and Waldor 2002), particularly plasmids, since a number of authors consider concepts relevant to phage-VF gene coevolution when considering the evolution of gene-MGE associations. Our fundamental aim, however, is the exploration of bacterial pathogenesis from the perspective of phage ecology. See Brüssow et al (2004) for review of the phage impact on the evolution of bacterial pathogens. See Chibani-Chennoufi et al (2004) and Breitbart et al (2005) for reviews of phage ecology as it applies to bacterial pathology, and Waldor et al (2005) for a general review of the phage impact on bacterial pathology. See Chibani-Chennoufi et al (2004) and Abedon (2006) for overviews of phage ecology in general.

## Enhancement of Gene Fitness

Selection acting on individual bacteria does not necessarily favor a continued gene-prophage or, more generally, a gene-MGE association. For instance, if a gene confers some benefit to a harboring bacterium, but an encoding prophage does not otherwise confer any advantage to the same bacterium, then over time we would expect evolution to favor a deletion of the prophage sequences accompanying a given gene (Ziebuhr et al 1999; Lawrence et al 2001; Desiere et al 2001; Brüssow et al 2004; Brüssow 2006) (see Bergstrom et al 2000 and Levin and Bergstrom 2000 for similar arguments vis-à-vis plasmids). Such deletions result in gene immobilization (a.k.a., “anchoring” or “fixation”) within a bacterial genome (Bergstrom et al 2000; Levin and Bergstrom 2000; Davis and Waldor 2002). Alternatively, selection for gene loss could act on prophage, purging or replacing phage genes that do not contribute to phage fitness. Such purging may be complicated, however, by size-dependent efficiencies of DNA packaging into phage capsids (Hendrix et al 2000; Hendrix and Casjens 2006). Contrasting these consequences of instability in the relationships between genes, prophage (or other MGEs), and bacteria, in this section we consider mechanisms based on advantages conferred to genes by mobility such that gene-phage or gene-MGE associations may be selectively *maintained.*

### 1. Gene survival via greater mobility

A gene found in association with a phage or other MGE could be maintained within a bacterial population simply if, as a consequence of that association, the gene is dispersed to new bacteria faster than the gene is deleted from individual bacteria (Eberhard 1990; Bergstrom et al 2000; Wagner and Waldor 2002). Enhanced mobility due to generalized transduction, in which a bacterial rather than phage gene is disseminated to new bacteria, is also thought to be capable of increasing the likelihood of gene maintenance within bacterial populations (Miller 2001). Since selection acting at the level of individual bacteria can favor the deletion of rarely utilized (Roth et al 1996) or otherwise costly genes, phage-mediated mobility could be particularly advantageous for genes that only rarely enhance bacterial fitness (Eberhard 1990; Levin and Tauxe 1996; Lawrence 1997; Levin and Bergstrom 2000), such as those coding for antibiotic resistance or for xenobiotic compound degradation (Eberhard 1990; de la Cruz and Davies 2000). Indeed, to provide an immediate fitness advantage to a recipient bacterium a gene would need to be *better* than the equivalent gene already found in that bacterium, a scenario that is unlikely given a lack of *a priori* coevolution between gene and bacterium, or alternatively (and more likely) provide or augment a novel or existing function (Lawrence and Hendrickson 2003). Lawrence (1997) argues similarly that the evolution of *non-essential* bacterial operons by a gradual building up of weakly selected, horizontally transferred genes is more consistent with novel rather than essential functions.

From the bacterial perspective, rarely utilized genes represent “a scattered reserve of ‘optional’ functions that enable populations or species to respond to new environmental contingencies” (Eberhard 1990) (see also Reanney 1976; Boyd and Brüssow 2002; Brüssow 2006). This occasional utility to bacteria—equivalent to phage “paying for dinner” (Levin and Bergstrom 2000), “paying their rent” (Adhya et al 2005) or, simply, “rent” (Hendrix 2005), or appeasing a bacterium with a “peace offer” (Desiere et al 2001) (also Hendrix et al 2000; Boyd and Brüssow 2002; Brüssow 2006; Hendrix and Casjens 2006)—would then help define the equilibrium frequency within a population at which a gene survives. In other words, phage may offset the metabolic and other costs associated with prophage harboring within a bacterium by supplying that bacterium with functions that the bacterium would otherwise not possess, though it is questionable whether these additional functions would be useful under all environmental circumstances. Lysogens nevertheless should display less of a disadvantage (or perhaps even display an advantage; Edlin et al 1975; 1977; Lin et al 1977) when prophage carry these peace offers, thereby selecting for peace offer retention within phage genomes. As Hendrix (2005) describes the situation, prophage deletion is more difficult, from a bacterial fitness perspective, given the embedding within a prophage of lysogen-fitness enchancing genes. Phage-associated peace offers can range from phage-encoded immunity functions against subsequent phage infection to factors that enhance bacterial virulence, ie, VFs.

If mobility selects for gene-MGE associations, then should we expect preferential MGE association with certain types of genes? In one approach to answering that question, Levin and Bergstrom (2000) argue that the need for peace offers (or being “nice” to their hosts as they describe the concept) likely is greater the less efficient (or less effective) the method of MGE horizontal transfer. Prophage— with robust mechanisms of release from bacterial hosts (eg, via phage-induced bacterial lysis; Young 2005) along with effective mechanisms of acquisition of new bacteria (ie, via virion adsorption)—possess a highly efficient means whereby genes may be horizontally transferred between bacteria. We therefore might predict a reduced bacterial utility associated with phage-encoded VFs compared to, for example, plasmid-encoded VFs. Stated more strongly, it is conceivable that phage-encoded VFs could even be detrimental to the fitness of harboring bacterial lysogens especially if phage-virion mediated horizontal transmission can make up for VF fitness costs associated with prophage-mediated vertical transmission.

### 2. Genetic hitchhiking on more-fit bacterial lineages

The above-posited mechanism(s) suggest that some combination of gene mobility and infrequent selection could be sufficient to maintain gene-prophage (or gene-MGE) associations. Note that we made no assumption of fitness differences between bacteria receiving MGE-associated genes, except *following* prophage (or MGE) acquisition. By contrast, there exists a second gene-centered explanation for maintenance of gene-MGE associations—one that presumes differences in bacterial fitness prior to, rather than following, prophage or MGE acquisition (Eberhard 1990; Turner et al 1998; Bergstrom et al 2000; Levin and Bergstrom 2000). Here the idea is that some bacterial lineages are inherently more fit within a given environment. Those lineages that are less fit may be driven to extinction, or, at least, will be at a competitive disadvantage to more-fit lineages. It stands to reason that genes that hitchhike on more-fit bacterial lineages can display a higher fitness than genes associated with less-fit lineages. Since MGE-associated genes are more mobile than other bacterial genes, the likelihood of a gene becoming associated with higher-fitness bacterial lineages should be greater than that of bacterial genes not associated with MGEs. More to the point, the likelihood of a gene being associated with at least one bacterial lineage that is not subsequently driven to extinction, eg, as via periodic selection (Koch 1974; Lenski 1984; Tiedje et al 1989), should be greater given greater gene mobility.

Still, from the bacterium’s perspective, once genes have been acquired there should be little incentive to retain, via this hitchhiking mechanism, a gene-prophage (more generally, gene-MGE) association (Davis and Waldor 2002): Not only are prophage potentially harmful to harboring bacteria—eg, due to metabolic demands associated with replicating prophage DNA as well as lysis-mediated virion release following prophage induction (Lawrence et al 2001; Brüssow 2006)—but so also should be the sharing, via prophage-mediated transfer, of useful genes with competing bacterial lineages. Thus, even though it is a reasonable argument that bacterial pathogens may be pieced together by their sequential acquisition of prophage and other MGE-encoded VFs (Brüssow 2006), such an argument does not explain why the sequence of the carrying prophage should be retained, over long time spans, within a given bacterial lineage.

### 3. Gene escape from immune surveillance

If greater bacterial invasiveness results in an increase in bacterial susceptibility to a host’s immune response, and acquisition of an MGE-encoded VF gives rise to greater bacterial invasiveness (eg, Boyd 2005), then VF acquisition could ultimately result in a decline in the fitness associated with a specific bacterial serotype within an infected animal. Greater invasiveness, as a consequence of opening new niches for bacterial propagation, nevertheless could be equated with greater bacterial fitness over shorter time frames (eg, Levin and Bull 1994; Frank 1996). An MGE-associated VF gene could thereby enhance bacterial invasiveness/short-term fitness as a peace offer. Ultimately, however, if greater invasiveness gives rise to greater antibacterial specific immunity, then the same VF could contribute to an animal-host specific decline in the fitness of a VF-harboring bacterial lineage.

If the bacterium’s antigenicity is bacterium encoded rather than bestowed by a MGE-encoded gene, then MGE association would allow the VF gene to relatively easily acquire alternative bacterial hosts that are not currently recognized by an animal’s immune response. To our knowledge this specific scenario has not been observed within a single animal over the course of bacterial infection. Over a community of animal hosts, however, such a scenario would be equivalent to VF horizontal transfer to bacteria displaying a wide range of antigenic variation (Breitbart et al 2005) and would constitute a variation on the hitchhiking scenario described immediately above. As a consequence, an animal’s immunity to one bacterial serotype would not necessarily preclude subsequent VF utility within the same animal. Of course, this scenario of association with antigenically distinct bacterial hosts should result in advantages to any genes that are not specifically involved in modifying bacterial antigenicity. Such strategies, however, may be especially relevant to genes, such as VF genes, that, by effecting bacterial invasiveness, are especially proficient at provoking anti-bacterial immune responses.

### 4. Gene extrabacterial survival

Wagner and Waldor (2002) suggest that “virulence genes encoded by phages may withstand environmental exposure better than those encoded by bacteria,” a hypothesis that implies selection for linkage between VF genes and the extracellular durability of phage virions as mediated by the phage morphogenesis genes (see also Muniesa et al 1999; Miao and Miller 1999; Breitbart et al 2005). This latter possibility, however, begs the question of whether or to what extent biases exist whereby some genes tend to benefit from this extracellular durability (or any other positive aspect of phage encoding) while others are “content” to remain *un*associated with phage genomes. One possibility is that it is those genes that are less-often employed by bacteria (ie, as discussed above) that are less readily retained within the bacterial genomes by natural selection. To avoid extinction, such less-employed genes presumably can more readily benefit from, and indeed may require, some combination of MGE-mediated population expansion and virion-mediated longer-term survival. These explanations, however, return us to the concept of peace offers and the question of whether or to what extent intermittent or non-essential gene utility to bacteria can select for ongoing VF association with prophage.

### 5. Faster gene evolution

Phage-mediated mobility can result in faster gene evolution through increased rates of mutation, recombination, or adaptation (Villarreal 2001). Implicit to an advantage associated with more-rapid gene evolution are assumptions that genetic variation can increase a gene’s usefulness such as could occur given significant environmental heterogeneity, eg, genetic variation as a means of immune-system evasion. Indeed, in some instances lysogenic conversion has been shown to result in modification of a bacterium’s antigenicity (Boyd and Brüssow 2002; Brüssow et al 2004). Note that positing fitness advantages given greater gene variation is not a claim that phage retain certain genetic sequences — such as could come into being as a consequence of random mutation — because of an anticipation of future utility. Instead, the argument is that the utility of a gene may be more readily enhanced given a more-rapid exploration of gene sequence space. Note that enhanced exploration of sequence space mostly occurs post gene transmission to a new bacterial lineage (with recombination as a variation-generating mechanism possibly an exception), and therefore does not benefit the previously harboring bacterial lineage so much as the recipient.

The first of these enhanced gene-evolution hypotheses is presented by Bishai and Murphy (1988) (see also Eberhard 1990). They suggest that MGEs, such as induced prophage, go through more rounds of replication than their host bacteria and therefore are more susceptible to mutation than chromosome-based genes. Alternatively, given their smaller genome size and proportionally lower DNA polymerase precision (Drake 1991), the per-polymerization, per-nucleotide mutation rate of phage genes ought to be greater, given virion production, than the mutation rate associated with equivalent bacterial genes. One can also posit a greater potential for changing a gene’s dosage given association with MGEs, whether within multicopy plasmids, tandemly amplified transposons, or multiple prophage (Eberhard 1990; McDonough and Butterton 1999). Given multiple copies it may also be possible to attain a pseudogene status in which evolving genes are less bound by requirements for ongoing functionality. Lawrence (2001), however, argues that we should expect natural selection to counter pseudogene accumulation by favoring bacterial lineages that have deleted non-essential and, especially, bacteria-detrimental genetic sequences.

Genes associated with MGEs may also experience more recombination events with related genes than do chromosome-bound genes (Eberhard 1990). For example, many phage genomes are mosaic in structure. Mosaicism implies that these genomes have been pieced together, over evolutionary time, via recombination events (Hendrix et al 1999; Canchaya et al 2003; Hendrix 2005) such as between a prophage and a superinfecting virus (Lawrence et al 2002) or—given polylysogeny (multiply lysogenized bacteria)—within “phage factories” (Ohnishi et al 2001). In addition to modifying individual structural genes, recombination can change the immediate genetic context of phage-associated genes (Mirold et al 2001; Krylov 2003). A great deal of genetic variation can be found, for example, among prophage displaying otherwise similar exotoxins (Mirold et al 2001; Recktenwald and Schmidt 2002), and a prophage-encoded gene presumably can have its expression modified by re-combinant changes in phage sequence external to the structural gene (LeJeune et al 2004). Variation in bacterium or environmental backgrounds may also occur as phage move from host to host (eg, as is probably observed for plasmid-born genes; Eberhard 1990) (see also Desiere et al 2001). Given MGE carriage it therefore seems reasonable that there exists greater potential for heterogeneity in the *context* of gene expression and evolution.

## Enhancement of Bacterial Fitness

While mobility by itself may confer a selective advantage on a gene, such enhancement of a gene’s fitness does not necessarily select for gene retention within a phage genome. Either a prophage-carried gene is useful to a prophage-infected bacterium, with the *prophage* at least potentially expendable, or the carried gene is not often useful to the phage or the bacterium, thereby rendering the *gene* expendable. Consistent with the Selfish Operon Model of Lawrence (1997; 2000), we therefore speculate that ongoing prophage retention of specific VF genes is more common if there is enhancement of phage fitness (that is, rather than or in addition to enhancement of bacterial fitness; Lawrence and Hendrickson 2003), or if a gene’s utility to its harboring bacterium is enhanced given prophage association. All subsequently considered scenarios invoke one or both of these criteria.

There are at least three mechanisms that could, by enhancing bacterial propagation, contribute to maintenance of an association between VF genes and prophage: (i) expression of phage genes leading to an increase VF utility to bacteria, (ii) virion packaging of VF genes to promote community-wide mechanisms of bacterial pathogenesis (ie, by disseminating an effective toxin dose), and (iii) phage release associated with VF expression that results in a numerical reduction in bacteria capable of directly competing with the unlysed kin of induced lysogens (ie, by lysogen “allelopathy”). These are mechanisms 6, 7, and 8 as presented in Table 1. In the two latter cases the resulting gene mobility may be considered, from the bacterium’s perspective, as an unintended consequence of utilization of phage to achieve otherwise unrelated ends: Either toxin genes are broadcast to normalflora bacteria using replication-competent phage (ie, in the dissemination of an effective toxin dose) or replication-competent phage are employed as bacteriocin equivalents (ie, as in lysogen allelopathy). Consequently, these latter mechanisms may be effectively viewed more from the perspective of bacterial populations or communities rather than solely from the perspective of the fitness associated either with individual genes or individual bacteria (ie, as addressed in mechanisms 1 through 5, Table 1).

### 6. Epistasis linking VF and phage genes

If the selective benefit associated with a gene is either enhanced or is fully dependent on the presence of additional MGE-associated genes, then selection may favor continued association (including via genetic linkage) of gene with MGE (Lawrence 1997). Diphtheria toxin production by *Corynebacterium diphtheriae*, for example, can be enhanced by the induction of the encoding prophage (Wagner and Waldor 2006). Reliance on additional phage genes for VF expression is seen with the Shiga toxin genes of *Escherichia coli*, which depend upon induced bacterial lysis for toxin release into the extracellular environment (Plunkett et al 1999; Wagner et al 2002; Davis and Waldor 2002). Lending credence to this idea of genetic linkage, Shiga toxin genes are co-regulated and approximately co-located with lambdoid lysis genes (Neely and Friedman 1998). Furthermore, in *Shigella dysenteriae* strain 1— which otherwise has completely lost phage-associated sequence—phage lysis genes are not only present but are genetically linked to the Shiga toxin genes, with all located within a single ~5000 bp region of the bacterial chromosome (McDonough and Butterton 1999; Davis and Waldor 2002). Also consistent with an epistatic association between phage and VF genes, Wagner and Waldor (2002) suggest that “in situ prophage induction could help to explain when and where certain virulence factors are produced during the course of bacterial infection.” Note, however, that in circumstances where prophage induction is required for enhancement of VF utility, then the expressing bacterium—if killed by prophage-induced bacterial lysis—would presumably not receive a direct fitness benefit.

### 7. Dissemination of an effective toxin dose

In this section we consider how advantages associated with VF mobility might stem from disseminated expression of a VF gene. An assumption is made that the advantages associated with the expression of certain VFs (eg, exotoxins) may be shared among bacteria and furthermore that these bacteria may benefit from the collective production of a greater VF dose. To achieve this greater VF production it may be possible to disseminate the VF genes—via MGEs such as phages—throughout communities of normal-flora bacteria. This effect may be described as the dissemination of an effective VF (or toxin) dose (Bishai and Murphy 1988; Plunkett et al 1999; Gamage et al 2003). From Bishai and Murphy (1988):

Phage conversion is a highly efficient means of rapidly disseminating the toxin gene within a nonimmune host. In the case of *C. diphteriae* the bacteria double every hour *in vitro,* but in that same hour a lytic corynephage can produce 30–60 converting phages. Nontoxigenic *C. diphtheriae, S. pyogenes, S. aureus,* and *E. coli* are all frequent if not constant members of the normal human flora. For these autochthonous organisms, *in situ* phage conversion represents a means of rapid spread of the toxin gene once a susceptible host has been infected with just a few toxinogenic organisms.

If phage-associated VF genes are to be disseminated, then prophage induction and virion adsorption and infection must occur *in situ.* Such interactions between phage and various bacterial pathogens can indeed occur over the course of at least experimental infection of animal hosts (reviewed in Table 3 of Breitbart et al 2005).

Smith (2001) provides a conceptual variation on dissemination of an effective toxin dose that is based on two assumptions: (i) That VF production may be costly to expressing bacteria and (ii) that factors released can benefit more bacteria than just the immediate producers. Given gene mobility, Smith argues that costs of VF expression may be spread more equitably around a community of similar bacteria. Gene-MGE association, by this logic, would represent a means of forcing otherwise “freeloading” bacteria—bacteria benefiting from VF expression but that are not burdened by its expression—to do their fair share of the gene-expression work. Note that coordination between gene mobilization (such as following lysogen induction), gene expression, and gene utility could allow this forcing to occur over the course of active disease progression. Of interest, however, note that the scenario that Smith presents could also be interpreted from the perspective of selection for mobility acting on genes (mechanism 1) rather than selection acting on bacteria, with the products of selectively mobile genes exhibiting a *shared* utility within bacterial populations rather than a *rare* utility as highlighted previously.

We find phage dissemination of an effective gene dose to be a compelling explanation for gene-MGE association. This is true particularly from the perspective of disease progression. Nevertheless, we have two concerns with the formulation of this explanation. The first concern is that if one assumes that the bacterium is the unit of selection, then dissemination of an effective toxin dose can demand an assumption of inclusive fitness—the idea that the fitness of alleles can be a function of the combined fitness of related individuals rather than simply of the Darwinian fitness of individual organisms carrying and expressing the allele (Hamilton 1964a, 1964b) (see also Bossi et al 2003; Livny and Friedman 2004). Calls for inclusive fitness are plausible given maintenance of close associations among clonally related bacteria (Levin 1987) and occur by necessity if the bacteria providing the VF do not survive VF dissemination. Lack of survival of producing bacteria appears to be the case for Shiga toxi-genic *E. coli* (as discussed above) and also is potentially the case for two prophage-encoded *Streptococcus mitis* platelet-binding factors (Bensing et al 2001).

The second concern involves assumptions as to the efficiency of phage conversion. This conversion can require lysogeny (as in “lysogenic” conversion) or, more generally, can involve either lysogeny, chronic phage infection, or, alternatively, the lytic infection of some though not all bacteria in a population—all of which may be described as phage conversions (Barksdale and Ardon 1974). For lysogenic conversion to take place, a phage infection must be reduced to lysogeny. However, if reduction to lysogeny is very likely then “amplifying the number of virulence gene-encoding organisms in the body during infection” (Wagner and Waldor 2002) would be muted since it is lytic growth, not latent-period extensions such as lysogeny (Abedon 1989; Abedon et al 2001; 2003), that underlie the rapid phage population expansion that should fuel effective-dose dissemination. Thus, there exists an inherent conflict between effective-dose dissemination and reduction to lysogeny, but typically it is lysogens (including induced lysogens), rather than purely lytic phage infections, that are emphasized when studying the production of phage-linked VFs.

There is no reason to believe that either of these concerns is in any way fatal to hypotheses on the dissemination of an effective toxin dose. They do point, however, to the importance—for the sake of understanding disease progression—of the *in situ* characterization of (i) probabilities of lytic-lysogenic decisions, (ii) the potential of purely lytic infections to produce phage-encoded toxins, (iii) phage host range, and (iv) whether inclusive-fitness benefits really are sufficient to select for VF retention among bacterial pathogens. Some of these considerations have been addressed for *V. cholerae*, which potentially employs phage-mediated toxin dose dissemination in the course of infection (Lazar and Waldor 1998; Faruque et al 2001). Furthermore, expression and release of cholera toxin appears to bypass absolute requirements for inclusive fitness for continuation of the phage-VF association since cholera toxin (as well as phage release) does not require infected-bacterium lysis. Gamage et al (2003; 2004) similarly explore some of these issues, both *in vitro* and *in situ,* in terms of Stx production upon presumptively lytic infection of *E. coli*.

### 8. Lysogen allelopathy

Inefficient reduction to lysogeny can provide an alternative explanation of why toxin genes may associate with prophage, via a phenomenon that Stewart and Levin (1984) call lysogen allelopathic effects. The term allelopathy is borrowed from the ability of some plants to chemically block the growth of plant competitors. By *lysogen allelopathy,* Stewart and Levin imply that phage released from induced bacterial lysogens may block the growth of competing bacteria by infecting and subsequently killing those bacteria. By releasing phage just as toxins are released from induced lysogens, the remaining (uninduced) lysogens may accrue any benefit associated with toxin production just as potentially competing bacteria (eg, competing for nutrients or for space) are infected and then lysed by these phage.

Bacteriocins, including some defective prophage, could effect a similar competition-minimizing result (Eberhard 1990), but, unlike intact phage, are not equipped to disseminate an effective toxin dose. Note that allelopathic effects additionally can serve as a defensive measure since phage-attacked bacterial competitors should posses a reduced potential to display allelopathic effects in return. Extension and some experimental verification of these ideas on lysogen allelopathy can be found as applied to *Salmonella* (Bossi et al 2003) or *Streptomyces* (Smith 2006) ecology and as a novel approach (Platt et al 2003) towards the phage-therapeutic treatment of bacterial infections (Goodridge and Abedon 2003).

## Enhancement of Phage Fitness

In this section we reflect on the VF impact on phage propagation. We consider a positive impact to be something that results in an increased phage burst size, a decreased phage latent period (that is, a reduced phage generation time but without burst-size/fecundity cost; Abedon et al 2003), or an increased Darwinian fitness associated with phage progeny. This idea of a phage utility to phage-encoded VFs is not novel, though has not been rigorously explored. Levin (1996), for example, rhetorically asks how toxins might “confer an advantage” on bacteria “or the plasmids and *phages* that code for these toxins” (emphasis added). Wagner and Waldor (2002) suggest that phage or VF genes could serve as units of selection while Davis and Waldor (2002) remind us that “It is possible that prophage induction generally facilitates survival of the phage genome more than that of the bacterial host species.” Furthermore, Novick (2003) notes that “Mobile genetic elements are arguably selfish in that their evolution is driven by selective forces that operate on the elements themselves, independently of the host organisms within which they must of necessity reside.” Here we expand on these speculations.

### 9. Direct enhancement of phage fitness (eg, as by dual utility)

Perhaps VF expression can directly contribute to a greater burst size by the expressing phage (Bishai and Murphy 1988) or, alternatively, perhaps VF association with virion particles could be of dual utility to virion and bacterial pathogen (Bensing et al 2001; Wagner and Waldor 2002; Gentry-Weeks et al 2002; Boyd and Brüssow 2002; Davis and Waldor 2002; Brüssow et al 2004; Boyd 2005). Also consistent with a dual utility (though just barely) would be the retention of genes, including VF genes or even random pieces of DNA, that could contribute to phage fitness as a consequence of size-dependent efficiencies of phage-genome packaging in phage capsids (Hendrix et al 2000). Below we suggest an alternative to these various “dual-utility” hypotheses, suggesting instead that prophage-encoded VFs could enhance phage propagation through a more indirect route than a direct enhancement of infection fitness: modification of the extra bacterial ecosystems within which phage propagate.

### 10. Indirect enhancement of phage fitness (by ecosystem modification)

What is accomplished with VF expression? In general terms, it is ecosystem modification, particularly given VFs for which an effective dose may be disseminated throughout a bacterial community. Typically this dissemination would be of soluble factors such as exotoxins, and historically exotoxins have been the VFs most closely associated with phage (Wagner and Waldor 2002; Wagner and Waldor 2006). Disease is an obvious consequence of ecosystem modification (eg, despoilment of the human-or animal-body environment), and it is of interest that exotoxins capable of inducing toxinoses, diseases that can be caused solely by the presence of a single toxin, are almost all carried by “highly mobile genetic elements” (Novick 2003).

Ecosystem modification, unless representing some unintended consequence of VF expression, should provide some benefit to the expresser, whether this is to the encoding gene, the carrying phage, or the expressing lysogen. For the phage, fitness benefits may be measured in terms of bacteria successfully infected within a given ecosystem (ie, within-culture phage population growth) or in terms of successful phage dissemination to new ecosystems (between-culture phage transmission). Phage fitness therefore may be enhanced if exotoxins are capable of (i) increasing the health or metabolic activity of phage-susceptible bacteria (resulting, given phage infection, in increased phage burst sizes or decreased phage latent periods; Hadas et al 1997), (ii) by increasing the number or density of phage-susceptible bacteria available to free phage, (iii) by increasing free-phage diffusion rates within environments, or (iv) by increasing environmental mixing and therefore the likelihood that free phage will be transmitted to new locations that are inhabited by phage-susceptible bacteria.

Each of these criteria could be met by exotoxin production resulting in ecosystem modification. Exotoxin production, for instance, could lead to an increased availability within environments of nutrients—such as by Shiga toxin-mediated release of blood into the intestinal lumen (O’Loughlin and Robins-Browne 2001) or diphtheria toxin-mediated release of nutrients from killed human cells (Ewald 2004). These nutrient increases could result in a boost (i) in the health of or (ii) in the number or density of phage-susceptible bacteria. Exotoxin production could also result, for example, in a loosening of stools. This stool loosening could result (iii) in decreases in environment viscosity or (iv) in increases in environmental mixing, in either case resulting in increased rates or likelihood of virion dissemination. Thus, a phage that expressed an exotoxin could set up subsequently produced free-phage progeny for faster or broader environmental dissemination than a similar phage that did not produce the exotoxin. As follows we discuss in greater detail this potential for exotoxin production to enhance phage fitness.

#### 10a. Indirect enhancement of phage replication by ecosystem modification

For phage-encoded exotoxins that may be expressed independent of free-phage production, toxin production could be a means by which prophage prepare their environment for subsequent phage growth. This growth could be by means of lysogen division, implying equivalence between toxin production and provision of a peace offer (Brüssow et al 2004). Alternatively, an induced lysogen may produce more phage progeny (ie, display a greater burst size) if previous toxin production results in greater lysogen physiological health. In addition, and consistent with a central tenet particularly of Smith’s (2001) take on effective toxin-dose dissemination, bacteria not producing exotoxins also might benefit from exotoxin production. If these bacteria are phage susceptible, then—by increasing the density or health of these other bacteria—the fitness of phage progeny released from induced lysogens should also be enhanced. Thus, one can view exotoxins as impacting favorably on phage growth *in situ* whether phage are growing as stable lysogens, as induced lysogens, or as released virions.

This phage plus lysogen advantage collapses to just a lysogen (or bacterium) advantage if prophage are defective, which in turn collapses to assumptions only of inclusive fitness if the host bacterium also must be killed to effect exotoxin expression. In the first instance the prophage cannot take advantage of improvement in conditions for virion-mediated population growth because the prophage cannot produce functional virions. In the second instance, even the expressing bacterium cannot take advantage of improvements in conditions because the bacterium dies long before conditions improve. Note, then, the narrow distinction between inclusive fitness gains and phage gains: Inclusive-fitness gains are a function solely of the fitness gains made by intact bacteria that also harbor the toxin gene whereas non-defective prophage, by releasing free-phage progeny, additionally may benefit from gains made by phage-susceptible bacteria—bacteria that do not necessarily harbor the toxin gene. Thus, there is a similarity between these seemingly distinct gains, bacterium versus phage: Both, as posited, have to do with ecosystem modification such that bacteria, at least over the short term, display an enhanced potential for growth.

This view of phage modifying their environment in a manner that enhances bacterial growth is not necessarily limited to the impact of phage-encoded VFs. Indeed, phage-induced bacterial lysis alone should result in the release of previously sequestered nutrients, ie, those formerly associated with not-lysed bacteria, which could then become available to surviving bacteria [see (Weinbauer 2004; Abedon 2006; Miller 2006) for reviews of this concept as applied to aquatic environments]. Furthermore, lysed bacteria can release ectoenzymes (Morita 1997), which are normally intracellular hydrolytic enzymes that can break down extracellular substrates that otherwise may be unavailable as a nutrient source to neighboring bacteria. Thus, we can describe at least three routes toward bacteria nutrient acquisition that could become available as a consequence of phage action: lysing of neighboring bacteria, ectoenzyme action on otherwise unavailable substrates, and the degradation (or invasion) of eukaryote-host tissues via the action of phage-encoded VFs. All three mechanisms could benefit phage virions, lysogens, or bacterial infections, as well as neighboring bacteria that are not phage infected. The resulting benefits are equivalent in their location dependence to benefits accrued more generally by proximity to such soluble bacterial factors as exoenzymes or bacteria-encoded exotoxins.

#### 10b. Indirect enhancement of virion dissemination by ecosystem modification

Increasing physical movement, like increasing nutrient densities, could also benefit phages, resulting in selection for continued association between phage and movement-enhancing VF genes. Such movement could occur within as well as between bacteria-containing environments. Within bacteria-containing environments various phage-mediated modifications of virion movement are already known, particularly ones that facilitate phage movement to the surface of phage-susceptible bacteria. Enzymatic degradation of materials associated with the bacterium that a phage is adsorbing—with enzymes displayed, for example, by virion particles— are probably fairly common (eg, bacterial hyaluronidase; Baker et al 2002; Brüssow 2006) (also Hughes et al 1998; Scholl et al 2001). Alternatively, Broudy et al (2002) suggest that soluble phage-encoded enzymes could enhance phage diffusion *away* from lysed bacteria. They argue, in particular, that phage spd1 of *Streptococcus pyogenes* encodes a DNase that could aid in the breakdown of the bacterium chromosome following phage-induced bacterial lysis, reducing local viscosity and thereby aiding phage diffusion to new phage-susceptible bacteria. As a VF this DNase could also aid, as these authors note, in the liquefaction of pus [see Prevelige (2006) for a similar “diffusion-away” function attributed to the tailspike protein *of Salmonella* phage P22].

Phage-encoded VFs can also facilitate phage movement *between* bacteria-containing environments. The basic idea that physical movement between environments could select for VFs is summarized by Ewald (1994). That is, selection for increased pathogen virulence can result if the physical transfer of pathogens to new hosts is augmented by mechanisms that reduce former-host health. Diarrhea, for instance, is not necessarily an incidental consequence of pathogen infection but instead may serve as an efficient means by which enteric pathogens move their kind out of colons (Ewald 1994; Wagner and Waldor 2002; Davis and Waldor 2002; Brüssow et al 2004). Free-phage transmission between hosts (eg, from one colon to another) presumably could be augmented by the same mechanism. Thus, to the degree that diarrhea is a consequence of the expression of phage genes (eg, Livny and Friedman 2004), then one can envisage diarrhea as phage-mediated bacterial virulence that is selected precisely because it increases phage transmission to new ecosystems.

Diarrhea-like loosening of stools might also foster more-rapid mixing and phage diffusion within environments, thereby resulting in greater availability to phage of phage-susceptible bacteria within colons. Diarrhea, therefore, may be described as potentially aiding free-phage transmission to new bacteria *within* colonic environments as well as aiding free-phage or lysogen transmission *between* colons. In addition, it is conceivable that a localized stool loosening, such as to a degree that does not necessarily give rise to noticeable symptoms of diarrhea, could also locally aid phage dissemination within colons to susceptible bacteria. Thus, even given an absence of sufficient stool loosening so that phage (and bacteria) dissemination out of animals is enhanced, phage-encoded enterotoxins might still augment the fitness of encoding phage by means of local enhancement of virion dissemination.

## Infection Type and Exotoxin Utility

Perhaps the most striking conclusion from our analysis of mechanisms potentially retaining phage-VF gene associations is the relative dearth of mechanisms enhancing the fitness of individual bacteria. The sole mechanism we have identified is epistatic interactions between phage and VF genes (mechanism 6), but only given that linkage between those genes is a reasonable response to such epistasis and even then only if VF expression doesn’t coincide with death of the bacterial lysogens (eg, such death is necessary for Shiga toxin release). Instead, the bottom line for phage-VF gene association is that for such an association to be stably maintained there must be an advantage for the gene to remain in association with a functional phage genome. For a phage genome to be functional, from an ecological vantage, then phage-susceptible bacteria must be obtainable by released phage progeny such that the phage life cycle may be completed. We would argue that this functional requirement for bacteria to infect, and not just for phage production, could significantly constrain the evolutionary maintenance of associations between functional phage genomes and VF genes. That is, bacterial infections that are too invasive may be too homogeneous in terms of bacterial types present to provide temperate phages with susceptible bacterial prey, ie, ones not already lysogenized by the same phage. Too-invasive bacterial infections also may be too sealed off from the outside world to provide phages with a reasonable potential to disseminate to susceptible bacteria found elsewhere, other than via the normal bacterial portal of exit.

For these more sealed off infections we would envisage a closer correspondence between phage and bacterial interests, including mechanisms of dissemination. That is, there could be less reason for eukaryote-tissue modifying genes to be phage rather than bacterium encoded. Mucous membranes, by contrast, more likely carry normal-flora bacteria than do tissues that are deeply buried within animal bodies. It is of interest in terms of VF utility, therefore, that the three best studied of phage-encoded exotoxins—diphtheria toxin as produced by *Corynebacterium diphtheriae*, Shiga toxin as produced by Shiga toxigenic *E. coli* such as *E. coli* O157, and Cholera toxin as produced by *Vibrio cholerae*—are all associated with infections in which bacteria do not actively invade host tissues but instead are limited to colonization on mucous membranes (eg, Bloom and Boedeker 1996; O’Loughlin and Robins-Browne 2001; Foxwell et al 2004).

We suggest that the phage utility of VFs in general and the phage utility of exotoxins in particular may be found under those circumstances where modification of the eukaryote-host environment allows increased phage potential for acquisition of new bacterial prey. In these circumstances phage biology and ecology—eg, as by disseminating an effective toxin dose—may impact dramatically on resulting disease. To better understand not only the evolution of bacterial pathogens, but also the progression of bacterial disease, it therefore may be necessary to understand phage-encoded VFs not just from the perspective of expressing bacteria, but also in terms of the ecology of entire ecosystems. Of great pertinence, therefore, is determining which factors control phage impact *in situ* such as rates of lysogen induction, relative likelihoods of lytic versus lysogenic infection, phage burst size, phage host range, evolutionary dynamics of phage-associated genes, and the ability of purely lytic infections to produce bacterial toxins. Ultimately this knowledge of phage biology may promote not just a better understanding of those forces impacting phage-VF associations, but also a better understanding of prophage-mediated disease processes themselves.

## Figures and Tables

**Table 1:** Mechanisms selecting gene-prophage associations plus primarily benefiting entities.

	Selecting mechanism	Benefiting entities			
		gene	bacterium	bacter. pop.	phage
1	Gene survival via greater mobility	✓^a^	✗^b^	✗^c^	✗
2	Genetic hitchhiking on more-fit bacterial lineages	✓	✗	✗	✗
3	Gene escape from immune surveillance	✓	✗	✗	✗
4	Gene extrabacterial survival	✓	✗	✗^c^	—d
5	Faster gene evolution	✓	—e	—e	—
6	Epistasis linking VF and phage genes	—	✓^f^	✓^g^	—
7	Dissemination of an effective toxin dose	—h	✗^i^	✓^g^	—
8	Lysogen allelopathy	—	✗^i^	✓^j^	—
9	Direct enhancement of phage fitness	—	✗	✗	✓
10	Indirect enhancement of phage fitness	—	✗	✗	✓^k^

a In constructing this table we assume that if at any time a gene/allele contributes to the fitness of a harboring organism, then that gene is under positive natural selection. We indicate (with a tick) that the gene is a primarily benefiting entity only when gene benefits cannot be explained solely on the basis of enhancing the fitness of a or the harboring organism.

b We employ a cross to indicate a relative lack of benefits to the indicated entity. We do not distinguish in this table between selection acting on prophage versus uninduced bacterial lysogens.

c An alternative view is that gene mobility and extra-bacterial survival (ie, as within phage virions) can be beneficial to bacterial populations or communities by retaining a reserve of functions among bacteria, but we would view hypotheses based solely on such a perspective to be inherently group selectionist, especially given arguments that bacteria do not retain gene exchange mechanisms for the sake of gene exchange (Redfield 1993; Redfield 2001) and therefore likely do not contribute greatly to a retention of gene-prophage associations.

d Phage fitness-enhancing effects we consider solely in rows 9 and 10. We indicate absence of any comment on utility with the symbol —.

e Our arguments assume that faster gene evolution requires gene mobility (see ^c^, above).

f Such linkage can contribute to VF survival independent of bacterial fitness (as indicated in row 4), but in other cases may be considered to contribute to VF expression and thereby to bacterial fitness.

g Because VF deployment in some instances requires expressing-bacteria death, we also invoke “bacterial population” as a potential beneficiary of this deployment.

h Dissemination should contribute to gene mobility, but gene mobility we cover in rows 1–3.

i Since these mechanisms involve phage-induced lysis of phage-producing bacteria, the benefiting entity would be the bacterial population rather than the producing bacterium.

j Lysogen allelopathy we speculate can augment the fitness advantages associated with gene-prophage associations rather than representing a mechanism sufficient to explain such associations.

k This enhancement could occur in terms of phage replication or dissemination, and mechanisms which may enhance phage dissemination within or between environments could presumably also enhance bacterial dissemination within or between environments.
